# Targeting lymphangiogenesis to prevent tumour metastasis

**DOI:** 10.1038/sj.bjc.6603120

**Published:** 2006-04-25

**Authors:** M G Achen, G B Mann, S A Stacker

**Affiliations:** 1 Ludwig Institute for Cancer Research, Post Office Box 2008 Royal Melbourne Hospital, Victoria 3050, Australia; 2 Department of Surgery, The Royal Melbourne Hospital, University of Melbourne, Parkville 3050, Victoria, Australia

**Keywords:** inhibitor, lymphatic vessel, VEGF-C, VEGF-D, VEGFR-3

## Abstract

Recent studies involving animal models of cancer and clinicopathological analyses of human tumours suggest that the growth of lymphatic vessels (lymphangiogenesis) in or nearby tumours is associated with the metastatic spread of cancer. The best validated molecular signalling system for tumour lymphangiogenesis involves the secreted proteins vascular endothelial growth factor-C (VEGF-C) and VEGF-D that induce growth of lymphatic vessels via activation of VEGF receptor-3 (VEGFR-3) localised on the surface of lymphatic endothelial cells. In this review, we discuss the evidence supporting a role for this signalling system in the spread of cancer and potential approaches for blocking this system to prevent tumour metastasis.

The lymphatic vasculature is an important route for the metastatic spread of human cancer. Hence, in almost all carcinomas, the presence of tumour foci in lymph nodes is the most important adverse prognostic factor in apparently localised disease. Furthermore, inhibition of regional lymph nodal metastasis was associated with reduced distant organ metastasis in some animal models suggesting a pathway may exist for distant organ metastasis via the lymph nodes (e.g. see Krishnan *et al*, 2003). It was previously thought that lymphatic metastasis involved passage of malignant cells along pre-existing lymphatic vessels near a tumour, however, recent studies in animal models suggest that lymphangiogenesis can be induced by solid tumours and may promote tumour spread (for a review see Saharinen *et al*, 2004). Moreover, clinicopathological studies have revealed that lymphangiogenesis can occur adjacent to or within human cancers and that this correlates with metastasis to lymph nodes in some tumour types including head and neck cancer (Beasley *et al*, 2002) and cutaneous melanoma (Dadras *et al*, 2003) (Table 1). The location of tumour lymphatics may be an important issue for metastatic spread as some studies have indicated that intratumoural lymphatic vessels are nonfunctional and that peritumoural lymphatics are more important for this process (e.g. see Padera *et al*, 2002). The extensive experimental and clinicopathological studies of tumour lymphangiogenesis carried out over the past 5 years suggest that it may be a useful target for therapeutics designed to restrict cancer metastasis.

The best validated signalling system for tumour lymphangiogenesis involves the secreted glycoproteins VEGF-C and VEGF-D that signal via VEGF receptor-3 (VEGFR-3; also known as Flt4), expressed on the surface of lymphatic endothelial cells (Joukov *et al*, 1996; Achen *et al*, 1998; Veikkola *et al*, 2001). Vascular endothelial growth factor-C and VEGF-D have been shown to promote tumour lymphangiogenesis, the metastatic spread of tumour cells to lymph nodes and, in some cases, distant organ metastasis in multiple animal models of cancer (for a review see Saharinen *et al*, 2004). Furthermore, expression of these growth factors appears to correlate with lymph node metastasis in numerous common human cancers (for a review see Stacker *et al*, 2002a). Other protein growth factors that have also been implicated in tumour lymphangiogenesis are platelet-derived growth factors (PDGFs) (Cao *et al*, 2004) and VEGF-A (Hirakawa *et al*, 2005), however, the evidence for involvement of these molecules in tumour lymphangiogenesis is currently restricted to relatively few animal models and therefore requires analysis in a broader range of experimental models as well as extensive clinicopathological studies to correlate the expression of these molecules with metastasis in human cancer. In summary, the VEGF-C/VEGF-D/VEGFR-3 signalling system is currently the most attractive target for antilymphangiogenic therapeutics designed to restrict cancer metastasis, although it is likely that other validated targets will emerge in future. We therefore focus on the VEGF-C/VEGF-D/VEGFR-3 signalling system in this article.

## VEGF-C/VEGF-D/VEGFR-3 SIGNALLING AND TUMOUR METASTASIS

### Mechanisms of lymphangiogenic signalling

The lymphangiogenic growth factors VEGF-C and VEGF-D are synthesised as proproteins consisting of a central VEGF homology domain (VHD), containing receptor-binding sites, flanked by N- and C-terminal propeptides (Joukov *et al*, 1996; Achen *et al*, 1998). Subsequently, the propeptides can be proteolytically removed to generate mature forms consisting of VHD dimers. The full-length forms of both growth factors bind the lymphangiogenic receptor VEGFR-3 but the mature forms do so with greater affinity (Joukov *et al*, 1997; Stacker *et al*, 1999), suggesting that the degree of proteolytic processing of VEGF-C and VEGF-D may in part determine the extent of lymphangiogenesis induced by these proteins in a tumour. Once VEGF-C is processed to the mature form it acquires the capacity to bind VEGFR-2 (Joukov *et al*, 1997), a cell surface receptor tyrosine kinase thought to signal for angiogenesis. In the case of VEGF-D, the affinity for VEGFR-2 is increased approximately 290-fold by proteolytic conversion of the full-length to the mature form (Stacker *et al*, 1999). Hence the mature forms of VEGF-C and VEGF-D can induce angiogenesis (Cao *et al*, 1998; Rissanen *et al*, 2003). The enzymes known to process VEGF-C or VEGF-D are the serine protease plasmin (McColl *et al*, 2003) and members of proprotein convertase (PC) family, namely furin, PC5 and PC7 (Siegfried *et al*, 2003). Plasmin also cleaves some isoforms of the angiogenic growth factor VEGF-A, releasing them from the extracellular matrix or cell surface thus making them available for inducing angiogenesis (Houck *et al*, 1992). The PCs have also been implicated in activation of other growth factors involved in lymphangiogenesis or angiogenesis, such as PDGFs (Siegfried *et al*, 2005). Hence these proteases could play a broad role in regulating growth of lymphatics and blood vessels in cancer.

As is the case for receptor tyrosine kinases in general, activation of VEGFR-3 by its ligands VEGF-C or VEGF-D results in trans-phosphorylation of tyrosine residues in the cytoplasmic domains of the dimerised receptor (Dixelius *et al*, 2003). Such phosphorylation events are known to be critical for the regulation of receptor kinase activity and for receptor interactions with signal transduction molecules. Analysis of VEGFR-3 signalling in lymphatic endothelial cells in culture indicated that activation of VEGFR-3 alone was sufficient to protect the cells from apoptosis and to induce proliferation and migration (Makinen *et al*, 2001). At least some of these signals were transduced by protein kinase C-dependent activation of the p42/p44 MAPK signalling cascade and via induction of Akt phosphorylation, two important signalling systems known to be associated with cell growth and survival. Further aspects of the VEGFR-3 signalling pathways remain to be elucidated and may be more complex than originally thought given that VEGFR-3 has been reported to form heterodimers with VEGFR-2 (Dixelius *et al*, 2003).

### Lymphangiogenic signalling and tumour metastasis

A range of experimental studies in animal models demonstrated that the VEGF-C/VEGF-D/VEGFR-3 signalling axis can promote tumour lymphangiogenesis and the metastatic spread of tumour cells. One approach has involved expressing VEGF-C or VEGF-D in tumour cells and monitoring the effects on lymphatic vessels within or adjacent to solid tumours as well as assessing the degree to which tumour cells spread to lymph nodes. For example, expression of VEGF-C in breast cancer cells increased intratumoural lymphangiogenesis and metastasis to lymph nodes and lung after injection into mice (Skobe *et al*, 2001b). In other models, expression of VEGF-C in breast cancer cells promoted growth of tumour-associated lymphatics (Skobe *et al*, 2001a), that were in some cases infiltrated with tumour cells (Karpanen *et al*, 2001), and metastatic spread to lymph nodes (Mattila *et al*, 2002). In a transgenic mouse model of pancreatic cancer, expression of VEGF-C in tumour cells led to extensive lymphangiogenesis associated with the tumours and metastases in draining lymph nodes (Mandriota *et al*, 2001). Expression of VEGF-D in tumour cells of a mouse xenograft model promoted formation of intratumoural lymphatics, angiogenesis, tumour growth and metastasis to lymph nodes (Stacker *et al*, 2001). The lymphatic metastasis in this model was prevented by a neutralising VEGF-D antibody that blocked binding of this growth factor to both VEGFR-2 and VEGFR-3 (Achen *et al*, 2000; Stacker *et al*, 2001). Further, VEGF-D promoted tumour lymphangiogenesis and lymphatic metastasis in mouse models of pancreatic cancer (Von Marschall *et al*, 2005).

An alternative approach for assessing the role of the VEGF-C/VEGF-D/VEGFR-3 system in cancer metastasis has been to treat well-established animal models of tumour spread, that have not been genetically engineered to express VEGF-C or VEGF-D, with inhibitors of this signalling pathway and to monitor the effects on lymphatic and distant organ metastases. For example, the highly metastatic human lung cancer cell line NCI-H460-LNM35 was transfected with an expression construct for a soluble version of VEGFR-3 (soluble VEGFR-3) that binds VEGF-C and thereby inhibits signalling by endogenous VEGFR-3 (He *et al*, 2002). The resulting tumour xenografts in mice contained fewer intratumoural lymphatic vessels and there were less metastases in draining lymph nodes than for control tumours that did not express soluble VEGFR-3. Mice with control NCI-H460-LNM35 tumours (i.e. not expressing soluble VEGFR-3) were treated with recombinant adenovirus expressing soluble VEGFR-3 as an alternative inhibitory strategy – this also restricted lymph node metastasis (He *et al*, 2002). In another animal model, expression of a soluble VEGFR-3 in highly metastatic MT-450 mammary tumour cells suppressed metastasis formation both in the regional lymph nodes and the lungs of rats (Krishnan *et al*, 2003). More recently, a recombinant adeno-associated virus expressing a soluble VEGFR-3 was shown to inhibit lymph node metastasis in a melanoma model in mice (Lin *et al*, 2005) and inhibition of VEGF-C expression using small interfering RNA-mediated gene silencing reduced lymphangiogenesis, lymph node metastasis and spontaneous lung metastasis in a mouse mammary tumour model (Chen *et al*, 2005). However, inhibition of tumour lymphangiogenesis using a soluble VEGFR-3 was not sufficient to block lymph node metastasis in a mouse model of prostate cancer suggesting that lymphangiogenesis is not an essential requirement for lymphatic spread in all tumours (Wong *et al*, 2005).

Clinicopathological studies carried out over the past 7 years have reported that expression of VEGF-C, VEGF-D or VEGFR-3 can correlate with lymph node metastasis in human cancer, a topic that has been extensively reviewed in recent times (Stacker *et al*, 2002a, 2002b; He *et al*, 2004). According to some of these studies, VEGF-C expression in tumour cells correlates with lymph node metastasis in lung, colorectal and prostate cancer (for review see Stacker *et al*, 2004) and may be a prognostic factor in ovarian and cervical cancer (Ueda *et al*, 2002; Nishida *et al*, 2004). Vascular endothelial growth factor-D expression was reported to be associated with lymphatic involvement and a prognostic marker in colorectal cancer (White *et al*, 2002) and a predictor of poor outcome in epithelial ovarian carcinoma (Yokoyama *et al*, 2003). Interestingly, VEGF-D and VEGFR-3 were recently reported to be independent prognostic markers in gastric adenocarcinoma and the presence of VEGF-D was correlated with lymphatic metastases in this tumour type (Juttner *et al*, 2006). There have been many reports of human tumours in which tumour cells express lymphangiogenic growth factors, but it is important to note that tumour-associated inflammatory cells, such as macrophages, can also express VEGF-C and VEGF-D, and could thereby play an important role in tumour lymphangiogenesis (Schoppmann *et al*, 2002).

## TARGETING THE VEGF-C/VEGF-D/VEGFR-3 SIGNALLING AXIS

The approach for targeting the VEGF-C/VEGF-D/VEGFR-3 pathway that has been most extensively employed in animal models of cancer, but is yet to be tested in the clinic, is the use of soluble versions of VEGFR-3 that bind VEGF-C and VEGF-D, thereby inhibiting activation of endogenous VEGFR-3 (see previous section) (He *et al*, 2002; Krishnan *et al*, 2003; Lin *et al*, 2005). It is yet to be determined if a soluble VEGFR-3 construct binds VEGF-C and VEGF-D so that these growth factors are also unable to activate endogenous VEGFR-2 – if this were the case soluble VEGFR-3 would have the merit of preventing the contribution made by these growth factors to angiogenic signalling in cancer via VEGFR-2 as well as lymphangiogenic signalling via VEGFR-3. Soluble versions of cell surface receptors are already used in clinical medicine, such as Enbrel® (also known as etanercept), a recombinant soluble version of the p75 receptor for tumour necrosis factor, that is used to treat rheumatoid arthritis (for a review see Maini and Taylor, 2000).

Other potential antilymphangiogenic therapeutics that would not enter the cell includes monoclonal antibodies to VEGF-C and VEGF-D, which block binding to both VEGFR-2 and VEGFR-3. Such an antibody to VEGF-D has already been described (Achen *et al*, 2000) and was shown to block angiogenesis, lymphangiogenesis and lymph node metastasis in a VEGF-D-dependent mouse tumour model (Stacker *et al*, 2001). Monoclonal antibodies to VEGFR-3 that block the binding of VEGF-C and VEGF-D could also be effective. Such an antibody to VEGFR-3 has been shown to block regeneration of adult lymphatic vessels (Pytowski *et al*, 2005) but its effects in cancer have not been reported. This type of reagent would not block activation of VEGFR-2 by VEGF-C or VEGF-D, however, bispecific antibodies that bind both VEGFR-2 and VEGFR-3 (Jimenez *et al*, 2005) could possibly be used for this purpose. Monoclonal antibodies are well established as anticancer therapeutics, one example being an antibody to VEGF-A, known as bevacizumab or Avastin®, that is designed to restrict tumour angiogenesis and used to treat patients with metastatic colorectal cancer (Ferrara *et al*, 2004).

An alternative strategy to receptor constructs and antibodies involves small molecules that enter the cell such as inhibitors of the VEGFR-3 tyrosine kinase or of downstream signalling molecules. A range of small molecules has been shown to inhibit the VEGFR-3 kinase, in addition to multiple other kinases such as VEGFR-2, some of which are in clinical trials as anticancer therapeutics – these include BAY 43-9006, CEP-7055 and PTK787/ZK 222584 (for a review see Achen *et al*, 2005). The signalling mechanisms downstream of activated VEGFR-3 are inadequately understood to justify targeting this component of lymphangiogenic signalling at this stage.

**Figure 1:**
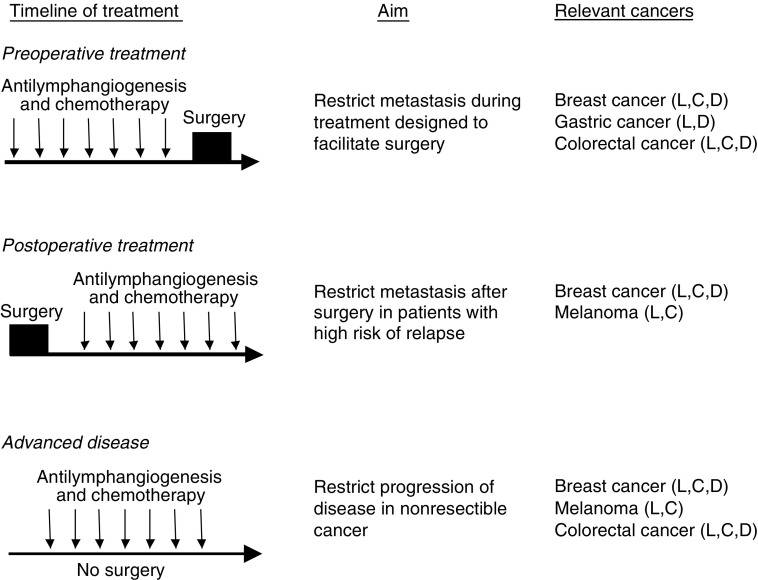
Clinical settings in which an antilymphangiogenic therapeutic agent, designed to restrict tumour metastasis, may be useful. Three scenarios are shown: top, during preoperative chemotherapy; middle, as part postoperative adjuvant therapy for patients with high risk for subsequent recurrence; bottom, during palliative systemic therapy of advanced tumours. In all cases it is envisaged that the antilymphangiogenic agent would most likely be effective in combination with cytotoxic chemotherapy. The course of each treatment is shown schematically to the left and the aim is listed in the central column. Human cancers for which these scenarios occur commonly are shown to the right with letters in parentheses denoting the following: ‘L’ that lymphangiogenesis has been associated with this tumour type; ‘C’ that for this tumour type VEGF-C expression has been reported to correlate with lymph node metastasis in at least one study; ‘D’ that for this tumour type VEGF-D expression has been reported to correlate with lymph node metastasis in at least one study (see Table 1 and Stacker *et al*, 2004).

How might an antimetastatic therapy, based on inhibiting tumour lymphangiogenesis, be utilised in the clinic? The management of most solid tumours involves surgical removal of the primary cancer to achieve local disease control and to restrict further metastatic spread. In cancers where effective systemic therapies are available, patients with tumour characteristics indicating there is a moderate to high risk of subsequent distant relapse are offered adjuvant therapy (Sun and Haller, 2005). This is usually in the form of cytotoxic chemotherapy, or some form of endocrine manipulation. Recently there has been intense investigation of the use of systemic therapies before surgery (Wolmark *et al*, 2001). This has been widely used to reduce the size of the primary cancer to facilitate surgery, for example by making a borderline resectible tumour more easily resectible, or to allow breast conservation for a tumour that would otherwise have required a mastectomy (Wolmark *et al*, 2001). More recently, there have been examples suggesting an improved cancer outcome with a preoperative approach (Malthaner and Fenlon, 2003). An antilymphangiogenic approach could be used in conjunction with conventional chemotherapy regimens in these scenarios (Figure 1), and may possibly reduce the incidence of local or distant recurrence. Treatment of advanced, nonresectible cancer remains inadequate in almost all cancers, and antilymphangiogenic agents may have a role in combination with other systemic agents in this scenario.

Several criteria should be met before introducing antilymphangiogenic agents to clinical trials. Reliable assays to detect the presence of VEGF-C, VEGF-D and VEGFR-3, and also to detect intra- or peritumoural lymphangiogenesis must be in place. Approaches for detecting these by immunohistochemistry, including identification of lymphatic vessels by staining for markers such as LYVE-1 (Dadras *et al*, 2003) and podoplanin (Renyi-Vamos *et al*, 2005), have been described in the clinicopathological literature and may require further development to become sufficiently robust. Initial trials should be performed on cancers in which lymphangiogenesis can be detected in a reasonable proportion of cases. Repeat biopsy and reassay after treatment is also important for the purposes of trials. While initial trials may be performed with patients with advanced disease, the neoadjuvant setting may yield the best results. Further work on tissue-banked specimens from breast, colorectal, esophago-gastric, lung and head and neck cancer specimens may identify the best candidate cancers, but ultimately it will require careful clinical trials with biological as well as clinical end points to assess this approach.

## CONCLUDING REMARKS

There is considerable experimental and clinicopathological evidence suggesting that tumour lymphangiogenesis is associated with the metastatic spread of cancer. Moreover, studies in experimental models of cancer have demonstrated that the VEGF-C/VEGF-D/VEGFR-3 signalling system is a key regulator of tumour lymphangiogenesis. This is further supported by clinicopathological analyses of human cancer showing that expression of VEGF-C and VEGF-D can correlate with lymph node metastasis in some human cancers. Numerous reagents that could be used to block this pathway already exist, including soluble VEGFR-3 protein constructs, neutralising monoclonal antibodies to VEGFR-3 and VEGF-D, and small molecule inhibitors of the VEGFR-3 kinase. Some of these reagents should also block the contribution of VEGF-C and VEGF-D to tumour angiogenesis via VEGFR-2, which could provide added benefit for patients. Hence, it is now appropriate to test this approach in the clinic. Some of the small molecule VEGFR-3 inhibitors are already in clinical trials, however, these trials are not specifically designed to monitor effects on metastasis. Indeed, clinical testing of an antimetastatic approach to cancer may require new strategies for trials so that the occurrence of cancer metastases can be monitored. Nevertheless, clinical testing of this approach is warranted in the near future.

## Figures and Tables

**Table 1:** Human cancers in which lymphangiogenesis has been observed

**Cancer**	**Type of lymphangiogenesis: IT or PT^a^**	**Is lymph node metastasis correlated with lymphangiogenesis?**	**Reference**
Head and neck cancer	IT and PT	Yes, with both IT and PT	(Beasley *et al*, 2002; Franchi *et al*, 2004)
Colorectal cancer	IT	Yes, with IT	(Kuroyama *et al*, 2005)
Cutaneous melanoma^b^	IT and PT	Yes, with both IT and PT	(Dadras *et al*, 2003)
Non-small-cell lung cancer	Predominantly PT	Yes, with PT	(Renyi-Vamos *et al*, 2005)
Gastric cancer	IT and PT	Yes, but type of lymphangiogenesis correlated with metastasis was not defined	(Nakamura *et al*, 2006)
Cancer of the uterine cervix	IT and PT	Yes, with PT only	(Gombos *et al*, 2005)
Breast cancer^b^	PT	No, lymph node metastasis correlated with tumour invasion of lymphatics, not with lymphatic vessel density	(Schoppmann *et al*, 2004)
Papillary thyroid cancer	IT (PT not analysed)	Yes, with IT	(Hall *et al*, 2003)

a IT denotes intratumoural and PT denotes peritumoural.

b There has been at least one report disputing that lymphangiogenesis occurs in this tumour type.
